# Thrombophilic risk factors and ABO blood group profile for arteriovenous access failure in end stage kidney disease patients: a single-center experience

**DOI:** 10.1080/0886022X.2021.2011746

**Published:** 2022-01-30

**Authors:** Sunnesh Reddy Anapalli, Harini Devi N., Pvgk Sarma, Lokanathan Srikanth, Siva Kumar V.

**Affiliations:** a Department of Nephrology, Sri Venkateswara Institute of Medical Sciences, Tirupati, India; b Department of Biochemistry, Sri Venkateswara Institute of Medical Sciences, Tirupati, India; c Department of Biotechnology, Sri Venkateswara Institute of Medical Sciences, Tirupati, India

**Keywords:** End-stage kidney disease, hemodialysis**;** hereditary thrombophilic factors, acquired thrombophilic factors, arteriovenous fistula failure

## Abstract

**Introduction:**

Thrombosis of fistula occurs most frequently in end-stage kidney disease (ESKD) patients receiving hemodialysis. However, the role of thrombophilia in arteriovenous fistula (AVF) failure has not been well established. Hence, this study was aimed at assessing the roles of hereditary and acquired thrombophilic factors in association with AVF failure among patients with ESKD undergoing hemodialysis.

**Methods:**

A cross-sectional study was conducted on 100 ESKD patients, of whom 50 patients with well-functioning AVFs with no fistula failures earlier were enrolled as Group 1, and 50 patients who have had AVF failure were enrolled as Group 2. The hereditary factors as factor V Leiden, factor XIII, prothrombin, and methylene tetrahydrofolate reductase and the acquired factors as lipoprotein (a), fibrinogen, homocysteine, and anticardiolipin antibodies IgG and IgM were studied.

**Results:**

Among the hereditary factors, no statistically significant difference was observed in relation to factor V Leiden and Prothrombin (*p* > 0.05). However, for factor XIII and methylene tetrahydrofolate reductase, a statistically significant difference was observed between patients with well-functioning AVFs and patients who have had AVF failure (*p* < 0.05). We found a statistically significant increase in all the acquired factors in patients who have had AVF failure in comparison with patients with well-functioning AVFs (*p* < 0.001). Association between ABO blood groups and thrombophilic factors showed significant association between factor V Leiden, anticardiolipin antibody IgG and IgM and ABO blood groups (*p* < 0.05), whereas none of the other thrombophilic factors showed significant association (*p* > 0.05).

**Conclusion:**

Thus, our study suggests significant role of acquired factors in causing AVF failure.

## Introduction

Vascular access is often considered an Achilles heel and represents a lifeline for end-stage kidney disease (ESKD) patients undergoing hemodialysis (HD) [1]. The majority of hemodialysis patients have either a native arteriovenous fistula (AVF) or synthetic arteriovenous graft (AVG) for vascular access [2,3]^.^ AVF failure is a problem for ESKD patients and one of the leading causes for morbidity and hospitalization, which have an impact on all-cause mortality and economic burden [4]. A fistula may thrombose usually within one or two years after the creation of access in 16–30% of ESKD patients due to certain risk factors [5]. Risk factors for AVF failure can be classified into demographic, clinical, haemodynamic, and technical factors. The demographic factors including age, sex, and ethnicity; the clinical factors such as heart disease, peripheral arterial disease, diabetes mellitus, obesity, etc.; the hemodynamic factors consisting of the size and diastolic function of vessels, blood flow, etc., and the technical factors such as the experience of the surgeon and the cannulation techniques employed during dialysis are the common causes for AVF failure [6]. Early thrombosis of fistula is mostly caused because of an inflow problem due to juxta-anastomosis stenosis or accessory vein, while the late thrombosis tends to be due to outflow stenosis. If either of these lesions are left untreated, they might result in the thrombosis of the fistula [7]. ESKD patients are usually in a substantial state of procoagulation that could manifest with a high incidence of cardiovascular events initiated by endothelium cell injury, leading to the formation of stenosis. Endothelium injury usually results from shear stress due to turbulent blood flow, mechanical trauma from venipuncture, and angioplasties [8,9]. As per recent observation by a laboratory study who studied effect of uremic serum on functional properties of human venous endothelial cells(VEC) and arterial endothelial cells(AEC) and reported VEC acquires the prothrombotic phenotype, whereas in AEC the inflammatory phenotype appears [10]. HD further stimulates coagulatory activity, which is accompanied by elevated levels of prothrombin fragments and thrombin–antithrombin-complexes [11]. Therefore, the literature focuses on the roles of various inherited and acquired thrombotic factors in association with thrombosis among patients with ESKD undergoing HD [12]. Till now, several gene polymorphisms have been studied in thrombophilia, which include the factor V Leiden gene mutation, prothrombin gene mutation, methylene tetrahydrofolate reductase (MTHFR) gene mutation, plasminogen activator inhibitor-1 mutations, factor H mutation, anti-thrombin, and protein C and protein S deficiencies [13,14]. Among these, factor V Leiden is considered the most commonly known genetic defect associated with venous thromboembolism. The factor V Leiden and prothrombin G20210A mutations are most commonly related to venous thrombosis, while increased levels of homocysteine are linked to a high risk of both arterial and venous thromboembolism [14,15]. These genetic defects when coupled with defects in the acquired factors can hasten renal thrombosis in ESKD [16]. One of the antiphospholipid antibodies found in the blood is anticardiolipin antibody, which is clinically associated with hypercoagulability. Anticardiolipin IgG and lupus anticoagulant are observed to be involved with thrombotic episodes, and their presence increases with HD [16,17]. Studies on both inherited and acquired thrombophilia in AVF failure are scarce among Indian patients with ESKD and the few results are contradictory [18]. Hence, the present study was undertaken to evaluate the roles of hereditary and acquired thrombophilic risk factors in ESKD patients with AVF failure receiving HD.

## Materials and methods

This was a mono-centric, cross-sectional observational study on 100 ESKD patients undergoing HD, who were selected during the study period 2017–18 from the Department of Nephrology. All 100 patients at the time of study were receiving intermittent HD using a functioning AVF. Of these 100 patients, Group 1 (*n* = 50) represented the controls who never had a history of fistula failure, and Group 2 (*n* = 50) included patients with a past history of fistula failure. Patients in Group 2 (*n* = 50) were further grouped as those with single AVF failure (*n* = 19) and those with multiple AVF failures≥ 2 (*n* = 31). Among the 31 multiple AVF failure subjects, two patients have had four AVF failures, seven patients had three AVF failures, and 22 patients had two AVF failures. This study was reviewed and approved by the institutional ethics committee (IEC No: 643/2017) and all participants provided written informed consent. The subjects included in the study were those with ESKD receiving HD treatment for at least six months through an arteriovenous fistula and had an age range of 18–80 years. All the AVFs were created at our hospital, in the theater, under the guidance of senior faculty in the Department of Urology. Concerning to the maturation and suitability of AVF for cannula initiation for MHD, the criteria applied were 12 weeks duration and the rule of six [19]. In the present study, fistula failure was considered in those cases in which AVF never went to the point that it can be used or it failed within the first three months of usage [20]. All AVF cannulations were done by trained and qualified dialysis technicians under the supervision of a senior dialysis technologist. All the patients and their personal caregivers were educated about AVF care before and after the procedure. MHD was performed thrice weekly for 4 h in addition to low flux conventional intermittent HD with heparin anticoagulation and bicarbonate dialysate. All the patients received erythropoietin (EPO). The exclusion criteria for subjects pertained to patients with unusable vascular accesses due to other reasons like infection besides thrombosis.

## Sample collection

Three mL of blood was collected in a vial containing ethylenediaminetetraacetic acid (EDTA), of which 2 mL was used for genetic analysis and 1 mL for biochemical analysis. Another 3 mL of blood was collected in a plain tube for biochemical analysis. Plasma samples were separated immediately, and the plain samples were allowed to clot, and then centrifuged at 3000 rpm for 15 min. Simultaneously, ABO blood grouping was performed.

Genetic analysis was carried out with real-time polymerase chain reaction (PCR). The blood sample was processed for the isolation of the whole blood genomic deoxyribonucleic acid (DNA) using phenol, known as the chloroform extraction method. The obtained DNA was confirmed through agarose gel electrophoresis and proceeds to PCR for Factor V Leiden, Factor XIII (Val34Leu), prothrombin (G20210A) and MTHFR (C677T) genes. The allele-specific mutated primers were designed for the genes that were responsible for thrombophilia. The PCR was performed in Eppendorf Master cycler nexus gradient using apparent conditions as mentioned in the table for respective genes, and the amplified products were documented with Vilber Lourmat’s gel documentation system [18].

## Biochemical analysis

Lipoprotein (a) and fibrinogen were estimated by immunoturbidimetry method, and anticardiolipin antibodies (IgG and IgM) were evaluated using enzyme-linked immunosorbent assay (ELISA) on ChemWell auto-analyser. Homocysteine was estimated with the enzymatic recycling method using UniCel DxC 600 auto-analyser, Beckman Coulter.

### Statistical analysis

Data distribution was assessed using the Kolmogorov–Smirnov test. Continuous variables were expressed as mean ± standard deviation (SD) and as frequency (number [%]) for categorical data. The comparison of occurrence of the hereditary thrombotic factors in the study groups were tested using the Chi-square test. The comparison of the means of biochemical parameters in the study groups were assessed using the independent samples t-test. Logistic regression analysis was carried out to identify the independent predictors of vascular access thrombosis such as age, gender, blood grouping, and the presence of diabetes. Further, binary logistic regression analysis was done to assess the association between ABO blood groups and thrombophilic factors. Statistical analysis was performed using Microsoft Excel spread sheets, SPSS software for Windows version 25 (SPSS Inc., Chicago, IL, USA). A p value of <0.05 was considered statistically significant.

## Results

In the present study, we investigated the roles of the hereditary and acquired thrombophilic factors in ESKD patients with normal and failed AVF undergoing HD (*n* = 100). Of them, 50 patients with well-functioning AVF who haven’t had fistula failures previously were enrolled as controls – Group 1, and 50 patients who have had AVF failure were enrolled as cases – Group 2. All the parameters studied had normally distributed data in at least one group and hence the data is presented with parametric statistical tools.

**Figure 1.**
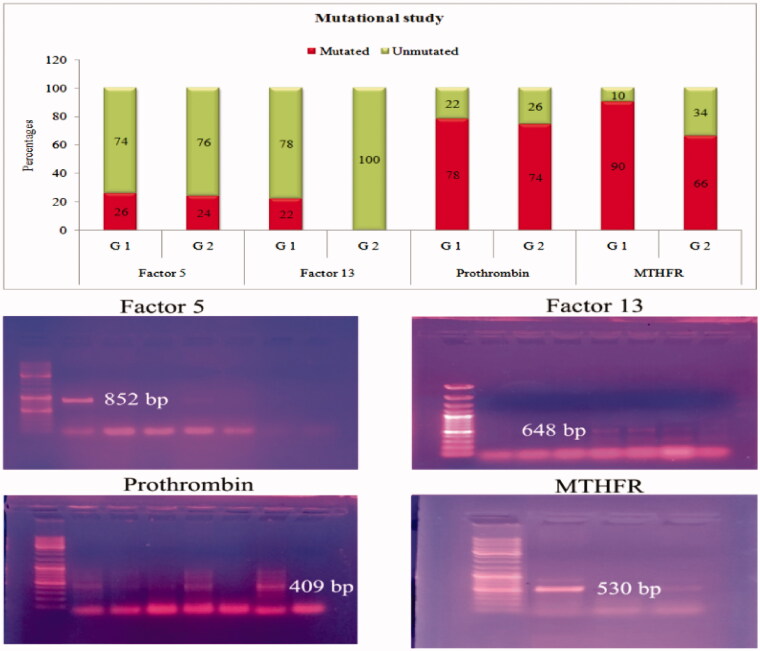
Allele specific mutational study Bar graph representing the percentages of mutated and unmutated genes among group 1 and group 2. The electrophoretic gels: representing the corresponding PCR products of factor5 (852 bp), factor 13 (648 bp), prothrombin (409 bp) and MTHFR (530 bp) genes in patients with mutation.

The demographic and clinical characteristics of hemodialysis patients are shown in Table 1. Patients from the A, B and AB blood groups were pooled as ‘non O’ blood group. ABO blood group frequencies among two groups (O and ‘non O’ blood group) were found to be not significantly different (*p* = 0.071^†^). Regarding the ABO blood group distribution, in the well-functioning AVF who haven’t had fistula failures group, 30 (50.0%) were O blood group and 20 (40.0%) were non O blood group. In the patients who have had AVF failure group 20 (40.0%) were O blood group, and 30 (50.0%) were non O blood group. Hemoglobin levels indicated statistically significant decrease in patients who have had AVF failure (*p* = 0.021). The occurrence of the hereditary thrombophilic factor gene mutations are represented in Table 2, Figure 1. In the present study, the hereditary thrombotic factors studied were factor V Leiden, factor XIII, prothrombin, and MTHFR. We observed that 13 patients with well-functioning AVFs with no fistula failures earlier and 29 patients who have had AVF failure had factor V Leiden mutations although not statistically significant (*p* = 0.817). In addition, we found that 11 patients with well-functioning AVFs had factor XIII mutations, whereas none had factor XIII mutations from patients who have had AVF failure, but statistically significant (*p* < 0.001). We also noted that 39 patients with well-functioning AVFs with no fistula failures earlier and 37 patients who have had AVF failure showed the prothrombin mutations; however, it was not statistically significant (*p* = 0.640). Furthermore, 45 patients had the MTHFR mutations in patients with well-functioning AVFs with no fistula failures earlier and 33 in patients who have had AVF failure, and it was found to be statistically significant (*p* = 0.004). However, from the subgroup analysis in patients who have had AVF failure for the occurrence of hereditary thrombotic factors between patients with single and multiple AVF failure, we observed that none of the hereditary thrombotic factors individually have statistically significant difference (*p* > 0.05). The comparison of the means of the acquired thrombophilic factors in the study groups is shown in Table 3. The acquired thrombophilic factors examined in this study were lipoprotein (a), fibrinogen, homocysteine, and anticardiolipin antibody (IgG and IgM) levels. In the present study, the acquired thrombophilic factors were found to be statistically significant in patients with well-functioning AVFs with no fistula failures earlier and patients who have had AVF failure were compared (*p* < 0.001). All the acquired factors showed a statistically significant increase in patients with AVF failure when compared with patients with well-functioning AVF (*p* < 0.001). The distribution of the acquired thrombophilic factors based on risk levels is shown in Table 4. All the acquired thrombotic factors in patients who have had AVF failure were found to be distributed above their cutoff, which was noted to be different and statistically significant when compared with normal levels. A subgroup analysis in patients who have had AVF failure was performed for the occurrence of the acquired thrombotic factors between patients with single and multiple AVF failure based on their respective cutoff levels (*p* < 0.001). Among the five acquired thrombotic factors, all except homocysteine were found to have statistically significant difference between patients with single and multiple AVF failure. Logistic regression analysis was performed in the study subjects with vascular access thrombosis as dependent variable and blood groups, the presence of diabetes, age, and gender as independent variables, as shown in Table 5. A significant association between vascular access thrombosis and ABO blood groups was observed with odds ratio OR (95% CI) 0.444 (0.199–0.989) (*p* = 0.047), whereas none of the other predictors showed significant association (*p* > 0.05). Further, logistic regression analysis was done to assess the association between ABO blood groups and hereditary and acquired thrombophilic factors as shown in Tables 6 and 7. A significant association between factor V Leiden and ABO blood groups was observed, whereas none of the other hereditary thrombophilic factors showed significant association OR (95% CI) 0.159 (0.028–0.902) (*p* = 0.038). Moreover significant association between anticardiolipin antibody IgG and IgM and ABO blood groups was observed OR (95% CI) 0.198 (1.021–1.406) (*p* = 0.027) for anticardiolipin antibody IgG and OR (95% CI) 0.834 (0.715–0.973) (*p* = 0.021) for anticardiolipin antibody IgM, whereas none of the other acquired thrombophilic factors showed significant association.

**Table 1.** Demographic and clinical characteristics of hemodialysis patients.

Parameters	Group1 (*n* = 50)	Group2 (*n* = 50)	*p* Value
Age (Years)	50.48 ± 13.69	49.82 ± 12.17	0.245**^b^**
Male:Female	35:15	36:14	0.826**^b^**
O Blood group	30 (60%)	20 (40%)	0.071**^b^**
Non O Blood group (AB,B &A)	20 (40%)	30 (60%)	
Diabetes mellitus	18 (36%)	19 (38%)	0.869**^b^**
HbSAg + ve	00	01	
HCV + ve	01	01	
HIV + ve	00	01	
Kt/V	1.21 ± 0.22	1.23 ± 0.26	0.236**^b^**
Dialysis vintage(months)	49.08 ± 10.02	49.32 ± 15.50	0.985**^b^**
S.Creatinine (mg/dl)	6.15 ± 2.31	6.37 ± 2.06	0.566**^b^**
S.Urea (mg/dl)	75.72 ± 12.75	87.36 ± 18.82	0.391**^b^**
Hemoglobin (g/dl)	10.97 ± 2.06	10.04 ± 1.87	**0.021^a^**

^a^Significant at the 0.05 probability level. ^b^NS-Not significant at the 0.05 probability level.

**Group 1**= ESRD patients with well functioning fistulas; **Group 2=** ESRD patients with failed AVF.

**Table 2.** Comparison of occurrence of the hereditary thrombophilic factors in the study groups.

Parameters	Group 1 (*n* = 50)	Group 2 (*n* = 50)	*p* Value
Factor V Leiden (n%)	13 (26%)	29 (58%)	0.817^b^
Factor XIII (val34leu) (n%)	11 (22%)	0 (0%)	<0.001a
Prothrombin (n%)	39 (78%)	37 (74%)	0.640^b^
MTHFR (n%)	45 (90%)	33 (66%)	0.004^a^

^a^Significant at the 0.05 probability level. ^b^NS-Not significant at the 0.05 probability level. Group 1= ESRD patients with well-functioning fistulas; Group2= ESRD patients with failed AVF.

**Table 3.** Comparison of means of acquired thrombophilic factors in the study groups.

Parameters	Group 1 (*n* = 50)	Group 2 (*n* = 50)	*p* Value
Lipoprotein (a) (mg/dl)	21.13 ± 7.58	30.13 ± 7.74	<0.001^a^
Fibrinogen (mg/dl)	270.13 ± 25.23	409.59 ± 58.09	<0.001^a^
Homocysteine (µmol/L)	12.14 ± 4.65	16.32 ± 6.08	<0.001^a^
Anticardiolipin antibody (IgG) GPL units	7.79 ± 3.28	24.80 ± 8.08	<0.001^a^
Anticardiolipin antibody (IgM) (MPL units)	6.49 ± 2.34	20.05 ± 8.77	<0.001^a^

Data presented as mean ± standard deviation (SD) ^a^Significant at the 0.05 probability level. Group 1 = ESRD patients with well-functioning fistulas; **Group 2=** ESRD patients with failed AVF.

**Table 4.** Distribution of acquired thrombophilic risk factors in the study subjects.

Parameters	Cut off	Group 1 (*n*%)	Group 2 (*n*%)	Total (*n*%)	*p* Value
Lipoprotein (a) (mg/dl)	>20 (mg/dl)	21 (42%)	48 (96%)	69 (61%)	<0.001^a^
	< 20 (mg/dl)	29 (58 %)	2 (4%)	31 (31%)	
Fibrinogen (mg/dl)	> 400 (mg/dl)	0 (0%)	28 (56%)	28 (28%)	<0.001^a^
	< 400 (mg/dl)	50 (100%)	22 (44%)	72 (72%)	
Homocysteine (µmol/L)	> 15 (µmol/L)	13(26%)	26 (52%)	39 (39%)	0.008^a^
	< 15 (µmol/L)	37 (74%)	24 (48%)	61 (61%)	
Anticardiolipin antibody (IgG) GPL units	> 10 GPL units	14 (28%)	50 (100%)	64 (64%)	<0.001^a^
	< 10 GPL units	36 (72%)	0 (0%)	36 (36%)	
Anticardiolipin antibody (IgM) (MPL units)	>15 MPL units	2 (4%)	39 (78%)	41 (41%)	<0.001^a^
	<15 MPL units	48 (96%)	11 (22%)	59 (59%)	

^a^Significant at the 0.05 probability level. ^b^NS- Not significant at the 0.05 probability level. Group 1= ESRD patients with well functioning fistulas; Group 2= ESRD patients with failed AVF.

**Table 5.** Logistic regression analysis in the study subjects with hemodialysis vascular access thrombosis as dependent variable and blood group, presence of diabetes, age and gender as independent variables.

Parameters	B	S.E.	Wald	*p* Value	Exp(B) (odds ratio)	95% C.I Lower bound	95% C.I Upper bound
ABO Groups	−0.812	0.409	3.951	**0.047^a^**	0.444	0.199	0.989
DM	−0.086	0.414	0.043	0.836**^b^**	0.918	0.407	2.067
Age	−0.004	0.016	0.062	0.803**^b^**	0.996	0.966	1.027
Gender	0.097	0.441	0.049	0.826**^b^**	1.102	0.464	2.615

^a^Significant at the 0.05 probability level. ^b^NS-Not significant at the 0.05 probability level.

**Table 6.** Logistic regression analysis in patients with thrombosed AVFs with ABO Groups as dependent variable and hereditary thrombophilic factors as independent variables.

Parameters	B	S.E.	Wald	*p* Value	Exp(B) (odds ratio)	95% C.I Lower bound	95% C.I Upper bound
Factor V Leiden	−1.840	0.886	4.311	**0.038^a^**	0.159	0.028	0.902
Prothrombin	1.184	0.822	2.077	0.150**^b^**	3.269	0.653	16.371
MTHFR	−0.951	0.743	1.640	0.200**^b^**	0.386	0.090	1.657

^a^Significant at the 0.05 probability level. ^b^NS-Not significant at the 0.05 probability level.

**Table 7.** Logistic regression analysis in patients with thrombosed AVFs with ABO Groups as dependent variable and acquired thrombophilic factors as independent variables.

Parameters	B	S.E.	Wald	*p* Value	Exp(B) (odds ratio)	95% C.I Lower bound	95% C.I Upper bound
Lipoprotein (a)	0.010	0.041	0.063	0.803**^b^**	1.010	0.932	1.095
Fibrinogen	−0.002	0.006	0.198	0.657**^b^**	0.998	0.987	1.009
Homocysteine	0.059	0.056	1.121	0.290**^b^**	1.061	0.951	1.184
Anticardiolipin antibody (IgG)	0.181	0.082	4.897	**0.027^a^**	1.198	1.021	1.406
Anticardiolipin antibody (IgM)	−0.182	0.079	5.336	0.021*	0.834	0.715	0.973

^a^Significant at the 0.05 probability level. ^b^NS-Not significant at the 0.05 probability level.

## Discussion

The maintenance of adequate vascular access is crucial for the survival of patient who is receiving HD. Thrombosis is the leading cause of AVF and graft failure, accounting for 80–85% of AVF access loss [3]. AVF failure occurs due to the interaction of several genetic and acquired thrombophilic factors [21]. However, there is a need for the identification of hereditary and acquired thrombophilic risk factors for the early diagnosis of thrombosis in patients with AVF failure. Hence, the present study was done to assess the roles of thrombophilic risk factors in relation to AVF failure to reduce these risks.

In the current study, the occurrence of hereditary and acquired risk factors for thrombophilia in HD patients was studied between the two groups. In our study, the factor V Leiden mutation was examined to find any association with vascular access thrombosis. In our study, the factor V Leiden mutation was found to be not statistically significant between the controls and cases (Group 1 & 2) (*p* = 0.817). These findings were similar to the previous study, which reported that the factor V Leiden mutation is not associated with vascular access thrombosis [22]. There was also another study that observed that the heterozygous carrier status for factor V Leiden did not appear to represent a risk factor for vascular access thrombosis in hemodialysis patients [23]. However, a recent study showed an increased risk of vascular access thrombosis in the carriers of the mutant FV (G1691A and A4070G) polymorphisms [24], and an Indian study demonstrated that the factor V Leiden mutation is the major causative factor in Budd–Chiari syndrome and portal vein thrombosis in the adult group, but it was not a major contributing factor in the pediatric group [25]^.^ In our study, the factor XIII mutation was examined to find any association with vascular access thrombosis. Factor XIII mutation was seen in 11 patients with well-functioning AVFs with no fistula failures earlier and none in patients who have had AVF failure that was found to be statistically significant (*p* < 0.001). This showed that the factor XIII mutation is not a significant thrombotic factor for vascular access thrombosis. A Human Genome Epidemology (HuGE) Review and meta-analysis provided evidence that the factor XIII val34Leu variant has a small but significant protective effect against vascular thromboembolism [26]. RF Franco and colleagues also observed that a mutation in the factor XIII val34Leu Variant protect against venous thromboembolism [27]. Another study showed that a common G-to-T point mutation (Val34Leu) in exon2 of the α-subunit of the factor XIII is strongly and negatively associated with the development of myocardial infarction [28]. In the present study, the prothrombin mutation was seen in 78% of patients with well-functioning AVF who haven’t had fistula failures and 74% of the patients who have had AVF failure, but it was not statistically significant (*p* = 0.640). In agreement with the present study’s findings, some studies did not find any significant association between the presence of the prothrombin gene mutation and vascular access thrombosis [27]. A study in India did not find any significant association between the prothrombin gene G20210A mutation and portal vein thrombosis [29]. However, a review study on hereditary thrombophilia reported that the prothrombin G20210A mutation is associated with an elevated risk of deep vein thrombosis [30]. In the present study, the MTHFR mutation was seen in 90% of the patients with well-functioning AVF who haven’t had fistula failures and 66% of the patients who have had AVF failure that was statistically significant (*p* = 0.004). A MEGA study did not indicate any association between mildly elevated homocysteine levels caused by MTHFR mutations and venous thrombosis [31]. Findings of a few other studies suggest that MTHFR C677T point mutations could be a risk factor for vascular access thrombosis in HD patients [32,33]. However, consolidating the findings of the present study, we could not find any significant effect of a single hereditary thrombotic factor on AVF failure, but the possibility of their effect in combination may need consideration as causative factors for arteriovenous fistula loss. The sample size is moderate, which might explain the reason for not finding any significant effect of individual hereditary thrombotic factor on vascular access thrombosis.

In the present study, increased lipoprotein (a) levels were found in patients who have had AVF failure when compared to patients with well-functioning AVF who haven’t had fistula failures (*p* < 0.001). The CHOICE study in hemodialysis patients showed that elevated lipoprotein (a) levels may be a risk factor for arteriovenous access complications among black HD patients [34]. A study based in India also showed an increased risk of arterial thrombosis with increased lipoprotein (a) levels [35]. Another study demonstrated that lipoprotein (a) levels were higher in patients with AVF failure than in the group with well-functioning AVFs [36]. These findings suggest an increased risk of vascular access thrombosis with an increase in lipoprotein (a) levels. In the present study, fibrinogen levels were increased in patients who have had AVF failure when compared to patients with well-functioning AVF who haven’t had fistula failures (*p* < 0.001). A case report of recurrent vascular access thrombosis in hemodialysis patients suggested increased fibrinogen levels along with other thrombotic factors [37]. Few other studies have also showed increased fibrinogen levels with other thrombotic factors in relation to vascular access thrombosis [38,39]. In the present study, homocysteine levels were found to be higher in patients who have had AVF failure when compared to patients with well-functioning AVF who haven’t had fistula failures, which was found to be statistically significant (*p* = 0.001). One study revealed that elevated homocysteine levels along with lipoprotein (a) levels causes increased risk for vascular access thrombosis and they are found to be associated with vascular access thrombosis [39]. Another study showed an independent association between hyperhomocysteinemia and AVF thrombosis in hemodialysis patients [13]. A prospective study reported hyperhomocysteinemia as the most common thrombophilia in both arterial and venous thrombotic events [40]. In the present study, it was observed that an increase in the levels of anticardiolipin antibody IgG and IgM in patients who have had AVF failure when compared to patients with well-functioning AVF who haven’t had fistula failures, and it was found to be statistically significant (*p* = 0.001). Another study found the incidence of arteriovenous thrombosis and the role of anticardiolipin antibodies in hemodialysis patients and found them to be associated with vascular access thrombosis [41]. A study in India showed an increased incidence of vascular access thrombosis in relation to antiphospholipid antibodies along with protein C and protein S deficiency [42]. The present study clearly observed that the acquired risk factors were found to be more participating for thrombophilia in patients with AVF failure patients. However, hereditary factors also noted to have impact on AVF failure as evidenced by their trend of increase in the vascular access failure group. These findings also suggest the monitoring of thrombophilic factors even before the creation of vascular access. If thrombophilic factors are found to be elevated, therapy to prevent thrombosis can be given as prophylaxis [43,44]. In the present study, we identified a statistically significant positive association between hemodialysis vascular access thrombosis and ABO blood groups (*p* = 0.047). However, there was no association between the presence of vascular access thrombosis with age, gender, and the presence of diabetes. Further, logistic regression analysis was done to assess the association between ABO blood groups and hereditary and acquired thrombophilic factors. In the present study a significant association between factor V Leiden and ABO blood groups was observed, whereas there was no significant association with other hereditary thrombophilic factors. The association between factor V Leiden mutation and vascular access thrombosis was previously reported [39,45]. However other studies did not observed association between factor V Leiden and vascular access thrombosis [22]. Moreover in the present study significant association between anticardiolipin antibody IgG and IgM and ABO blood groups was observed, whereas none of the other acquired thrombophilic factors showed significant association.

## Strengths of the study

Most studies in India have assessed either the hereditary or acquired factors. In contrast, both the hereditary and acquired thrombotic factors were studied in the present study.

## Limitations of the study

Confounding factors such as diabetes were also included. Allele frequency was not evaluated, which might be useful in identifying the mutation variant. Lastly larger sample sizes are needed to understand the patterns of risk factors.

## Summary and Conclusions

The present study findings conclude that, for hereditary thrombophilia factors, there was no observable difference between patients with well-functioning AVF who haven’t had fistula failures previously and patients who have had AVF failure . Moreover, we found a statistically significant increase in the acquired thrombophilia factors in patients who have had AVF failure in comparison with well-functioning AVF patients who haven’t had fistula failures, thus suggesting their potential role. The association between thrombophilic factors and ABO blood groups in the present study suggests that ABO groups showed a very similar association for both thrombophilic factors.

## Figures and Tables

**Table:** 

	Contributor 1	Contributor 2	Contributor 3	Contributor 4	Contributor 5
Concepts	√	√	√		√
Design	√	√	√		√
Definition of intellectual content	√	√	√		√
Literature search	√	√	√	√	√
Data acquisition	√	√	√	√	
Data analysis	√	√		√	
Statistical analysis	√	√	√	√	
Manuscript preparation	√	√			√
Manuscript editing	√	√		√	√
Manuscript review	√	√	√	√	√
